# The state of the art of extracellular vesicle research in protozoan infection

**DOI:** 10.3389/fgene.2022.941561

**Published:** 2022-08-12

**Authors:** Xinlei Wang, Jie Chen, Jingtong Zheng

**Affiliations:** ^1^ Department of Clinical Laboratory, The Second Hospital of Jilin University, Jilin University, Changchun, China; ^2^ Institute of Theoretical Chemistry, Jilin University, Changchun, China; ^3^ Department of Pathogenobiology, College of Basic Medical Sciences, Jilin University, Changchun, China

**Keywords:** extracellular vesicles, protozoan diseases, protozoa, exosomes, microvesicles

## Abstract

Protozoan diseases seriously affect the health of human beings, livestock and poultry and lead to high economic and medical costs. Extracellular vesicles (EVs) are membranous structures formed through biological processes that play important roles in immune regulation. Studies have shown that parasites transmit information to hosts through EVs to modulate host immune responses. The major roles played by EVs released from parasites involve facilitating parasitization of the host. In this review, we discuss relevant recently obtained data on EVs secreted by different kinds of protozoa, including their molecular mechanisms, and discuss the roles played by EVs in the occurrence and development of parasitic diseases.

## 1 Introduction

Protozoan diseases are among the most important diseases, with fast transmission rates and causes of great harm to animal husbandry (Turkeltaub et al., 2015). Protozoa can cause various important diseases, such as Chagas disease (Eberhard et al., 2022), leishmaniasis (Ribeiro-Dias and Oliveira, 2022), giardiasis (Hajare et al., 2022), trichomoniasis (Ibáñez-Escribano et al., 2022), and malaria (Kalkal et al., 2022). Currently, the prevention and treatment of protozoan disease mainly rely on medicinal plant extracts and chemically synthesized drugs, but resistance to certain drug treatments is an emerging problem (Dziduch et al., 2022). Therefore, gaining further understanding of the regulation of the host immune response to parasitic infection and its role in protozoan disease is an urgent need.

Secreted membrane-bound extracellular vesicles (EVs) are derived through different production pathways and exhibit biological characteristics and functions under physiological and disease conditions (Badimon et al., 2016; Puhm et al., 2021). The release of EVs is a universal biological process. As a carrier of membrane proteins, lipids and nucleotides for transfer to recipient cells (Jin et al., 2021), EVs show species specificity; moreover, they can exist in the extracellular environment and bodily fluids such as blood, urine, saliva and amniotic fluid, as well as in the medium supernatant of cells and parasites cultured *in vitro* (Caivano et al., 2015). Exosomes, microvesicles (MVs) and apoptotic bodies are also EVs (Suades et al., 2022). The difference in EVs is based on the difference in shape, size and/or formation (Buenafe et al., 2022). This paper mainly focuses on exosomes and MVs because most of these are produced in living cells. Currently, exosomes are thought to be intraluminal vesicles (ILVs) mainly formed by multivesicular bodies (MVBs) and released into the extracellular environment (Dong et al., 2022). In contrast, MVs are formed by budding directly from the plasma membrane (Zheng et al., 2022). Due to the special role played by EVs in protozoan parasite infection, in this review, we analyze the correlation between EVs and parasitic infection and differences in EV formation induced by different protozoan parasites (Table 1).

**TABLE 1:** Extracellular vesicles shed by different parasites and the associated signaling pathways.

Disease	Parasite	Types of extracellular vesicles	Regulatory factors of extracellular vesicles	Main pathogenic components	Reference
Chagas Disease	*Trypanosoma*	Exosomes and MVs	Temperature, pH value, parasite development stage and virulence	Tc-85, SMP, PGE 2, and PARP	Chambon et al. (1963); Alves et al. (1986); Abuin et al. (1996); Ba et al. (2010); D’Avila et al. (2011); Nogueira et al. (2015); Berger et al. (2018); Lovo-Martins et al. (2018); Lüscher et al. (2018); Choudhuri and Garg, (2020); Cronemberger-Andrade et al. (2020); Fehr et al. (2020); Velasco and Morillo. (2020); Dantas, (2021); Pedra-Rezende et al. (2021); Vasconcelos et al. (2021); Oliveira et al. (2022a)
Leishmaniasis	*Leishmania*	Exosomes and MVs	Temperature	GP63, CK1, GAPDH, and viruses	Razzazan et al. (2008); Hallé et al. (2009); Sachdeva et al. (2009); Silverman et al., 2010a; Barbosa et al. (2018); Atayde et al. (2019); Dong et al. (2019); de Carvalho et al., 2020; Hashemzadeh et al. (2020); Nogueira et al., 2020; Baker et al., 2021; Chan et al. (2021); Das et al. (2021); Dupin et al. (2021); Abdellahi et al. (2022); Oliveira et al. (2022b); Smirlis et al. (2022)
Giardiasis	*Giardia*	Exosomes and MVs	pH value and inducers	EVs	Solaymani-Mohammadi, (2022)
Trichomoniasis	*Trichomonas*	Exosomes and MVs	CaCl_2_, ESCRT, and temperature	Pmp, BspA, GP63, and Tetraspanin	(Henne et al., 2011; Twu et al., 2013; Olmos-Ortiz et al. (2017); Juan and Fürthauer, (2018); Nievas et al. (2018); Gavinho et al. (2020); Govender et al. (2020); Coceres et al. (2021); Molgora et al. (2021); Salas et al. (2021); Schumann and Plasner, (2021); Ong et al., 2022)
Amebiasis	*Entamoeba*	Exosomes and MVs		Argonaute 2 and TSN	(Calixto-Gálvez et al., 2011; Foda and Singh, (2015); Cázares-Apátiga et al. (2017))
Granulomatous amoebic encephalitis	*Acanthamoeba*	Exosomes and MVs	Culture media	ASP	Huang et al. (2017); Gonçalves et al. (2019); Lin et al. (2019)
Malaria	*Plasmodium*	Exosomes and MVs	Parasite development stage	ABCA1	Jensen et al. (2020); Peters et al. (2022)
Neosporosis	*Neospora*	Exosomes	Parasite development stage	EVs	Li et al. (2018)

## 2 Phylum sarcomastigophora, class zoomastigophorea

### 2.1 Chagas disease—*Trypanosoma cruzi*

Chagas disease is a zoonotic parasitic disease caused mainly by *T. cruzi* (Eberhard et al., 2022). Its main vector is blood-feeding triatomine bugs (Eberhard et al., 2021). In recent years, with the effective control of these vectors, other transmission methods, such as oral transmission, organ transplantation and blood transfusion, have gradually attracted attention. The World Health Organization (WHO) reported that Chagas disease has affected approximately eight million people worldwide (Campetella et al., 2022). Most people are asymptomatic and present no clinical signs, but as many as 30% of patients with Chagas disease are expected to develop heart, digestive or nervous system and potentially life-threatening disease many years after the initial infection (Sales et al., 2017). In 2019, the WHO established World Chagas Disease Day (14 April) to raise global awareness of the causes of Chagas disease and stimulate investment to fight it (https://www.who.int/).

#### 2.1.1 Characterization of *T. cruzi* extracellular vesicles

In 1991, Marinei first found that *T. cruzi* isolated from Chagas disease patients produces EVs (Gonçalves et al., 1991), and since then, researchers have become increasingly interested in EVs because of the key roles they play in intercellular communication. Studies have shown that the EVs released by *T. cruzi* are involved in the regulation of the host immune response to promote infection (D’Avila et al., 2021). On the basis of a size analysis, two kinds of EVs released by *T. cruzi* have been characterized: exosomes and MVs (Rossi et al., 2019).

EV shedding by *T. cruzi in vitro* can be regulated by many factors, such as temperature, pH value, parasite development stage and virulence (Vasconcelos et al., 2021). Studies have shown that although some kinds of parasites release more EVs at 26°C, *T. cruzi* EV shedding is most prolific at 4°C. Although the levels of EVs in the supernatant gradually increase at 37°C over time, the levels of EVs released by *T. cruzi* at 4°C is always more than twice those released at 37°C. In addition, with the increase in EV concentration, the level EV reabsorption by parasites also increases.

Moreover, the release of EVs is related to the pH value (Vasconcelos et al., 2021). The effect of different pH values on EV release was detected by incubating parasites in 37°C in medium containing 5% glucose for 2 h. These experiments revealed that the levels of the EVs released at pH 5 was more than 3- and 5-fold greater than those released at pH 7 and pH 9, respectively, but no change in the size of these EVs was detected. Because the pH value is closely related to parasite morphological changes and life cycle, the high peak of EV release from the parasite in the host body may follow a process similar to the invasion of mammalian cells by parasites (Pedra-Rezende et al., 2021). The infectious form of *T. cruzi* establishes a unique relationship with lysosomes in target host cells (Oliveira A. C. S. et al., 2022). Compared with many intracellular pathogens that avoid contact with lysosomes, *T. Cruz* needs to attach briefly to this acidic organelle to infect a host. The low pH environment of lysosomes promotes the entry of this parasite into the host cytoplasm, which is a key step in parasite development (Oliveira A. C. S. et al., 2022). Because infection can trigger the release of many EVs simultaneously, EVs play an important role in the invasion of mammalian cells by parasites. In addition, studies have shown that EVs released by *T. cruzi* can participate in invasion through a mechanism independent of lysosomes. In the non-lysosome infection pathway, invading parasites activate PI3K, resulting in Rab5 activation, which promotes the formation of phagosomes (Cronemberger-Andrade et al., 2020).

In addition, the composition of the EVs produced by *T. cruzi* differ by the virulence of the parasite strain. Lower protein content has been detected in EVs derived from the Y, Columbia and CL strains. The EVs of the YuYu strain show higher protein content (Nogueira et al., 2015). Studies have shown that the EVs of all *T. cruzi* strains (CA1, Y, YuYu and RA) contain Tc-85 antigen, a molecule related to cell invasion (Alves et al., 1986), but each strain expresses it at different levels (Abuin et al., 1996). In addition, differences in the activity of EVs secreted by different parasite strains have been detected in tissue incubated in culture with trypomastigotes (TCTs) in DMEM. Specifically, compared with those secreted by the YuYu strain, EVs secreted by the Y strain and those in patient plasma with chronic Chagas disease are highly reactive in α-Gal antibodies, suggesting that EVs recognize the α-Gal epitope (Nogueira et al., 2015).

#### 2.1.2 Effects of extracellular vesicles on chagas disease

Because *T. cruzi* can spread from infected sites to all host organs or tissues through lymph and blood vessels, the autonomic nervous systems I the heart (Velasco and Morillo, 2020), esophagus and megacolon (Dantas, 2021) are the most affected organs. In the early stage of infection, the control of parasites depends on innate immunity. Macrophages, as well as other innate immune cells, play key roles in regulating the host response to *T. cruzi* infection. Studies have shown that the EVs shed by parasites can lead to an increase in the number of parasites in the heart and produce a strong inflammatory response (Cronemberger-Andrade et al., 2020), suggesting the importance of EVs in Chagas disease.

The effects of EVs shed by different *T. cruzi* strain**s** (Y, Colombian, CL and YuYu) on regulation of immune responses (in the congenital and chronic infection phase) in C57BL/6 mice were evaluated. Studies have shown that EVs shed by different strains can cause high expression of inflammatory factors in macrophages (Nogueira et al., 2015; Cronemberger-Andrade et al., 2020). Among these EVs, CL- and YuYu- derived EVs induced higher levels of proinflammatory cytokines (TNF-α and IL-6) and nitric oxide (NO) through Toll-like receptor 2 (TLR2) in macrophages (Nogueira et al., 2015). However, different regulatory patterns were observed in the splenocytes of the chronically infected mice. Among *T. cruzi* strains, the Colombian strain induced the higher levels of proinflammatory cytokines. To test the importance of cytokine regulation, EVs from the YuYu and Colombian strains were used to evaluate the expression of intracellular cytokines in different cells. These studies showed that EVs from both the YuYu and Colombian strains induced cytokine production, and in particular, a high level of IL-10 was observed in CD4+ and CD8+ T lymphocytes (Nogueira et al., 2015). Various cytokines, such as TNF-α and IL-10, were produced in B lymphocytes. Moreover, dendritic cells produced TNF-α after stimulation with EVs. These results suggest that EVs may be determinants of immunopathological events not only in the early stage of infection but also in the chronic stage.

The influence of inflammatory factors on the occurrence and development of diseases may be related to certain protein components in EVs. Poly ADP ribose polymerase (PARP1), initially reported by Chambon et al. (1963), is a ribozyme closely related to DNA damage repair. Recently, PARP-1 has been shown to be mainly involved in DNA damage repair (Lüscher et al., 2018), cancer (Berger et al., 2018), and inflammatory damage (Fehr et al., 2020). Therefore, PARP-1 is closely related to various forms of pathogenesis. Moreover, *T. cruzi* EVs purified from parasite culture medium (called *Tc*EVs) and *T. cruzi* EVs purified from RAW264.7 cells and plasma of acute and chronically infected wild-type mice and Parp1^−/−^ mice (called *T*EVs) were detected. The results showed that the expression levels of the measured cytokines did not increase after 6 h of incubation with EVs in RAW264.7 cells; however, compared with those in the control group, the expression levels of cytokines such as TNF-α, IL-6 and IL-1β were dramatically increased at 18 hours and 48 h in culture. The EVs incubated with IFN-γ further increased the levels of TNF-α, IL-6 and IL-1β released by the RAW264.7 cells. Moreover, cells from infected muscle were found to be the main targets of these EVs. That is, compared with the EVs in control group cells, EVs in muscle cells greatly increased the TNF-α, IL-6 and IL-1β levels after 18 hours and 48 h of culture (Choudhuri and Garg, 2020).

Interestingly, the interaction between macrophages and *T. cruzi* EVs induced the production of prostaglandin E_2_ (PGE_2_). On the one hand, a high concentration of PGE_2_ decreased the gene expression of the proinflammatory cytokines IL-1 β and IL-6 (Lovo-Martins et al., 2018); on the other hand, it promoted the formation of macrophage liposomes, which participate in the immune escape mechanism triggered by *T. cruzi* (D’Avila et al., 2011). Therefore, EVs shed by *T. cruzi* into the extracellular environment seem to play a variety of regulatory roles in host cells.

When a parasite spreads from the infected site to the heart through lymph and/or blood vessels, the effect of the EVs released by the parasite on target cells is very important. The oxidized DNA encapsulated by EVs is an essential component that triggers macrophages in the heart to activate the PARP1-cGAS-NF-κB signaling pathway. When macrophages were incubated with DNA or protein in EVs, only the DNA components caused the reaction of proinflammatory cytokines in macrophages (Choudhuri and Garg, 2020). In addition, when *T. cruzi* was incubated with cardiomyocytes, although the role of the EVs was not specifically detected, the levels of inflammatory factors increased significantly, and PARP1 played a very important role in this increase (Ba et al., 2010). As a result, it has been suggested that oxidized DNA released by EVs worsens chronic inflammatory pathology in Chagas disease by activating inflammatory signaling pathways. Using small-molecule antagonists of the PARP1-cGAS signaling pathway may help to control chronic inflammation in Chagas disease.

Alves’ research group found that EVs exerted damage to the heart. Studies have shown that the addition of EVs before inoculation with *T. cruzi* caused more severe heart injury, shortened the time to the death of infected mice and increased the mortality of the infected mice. In addition, the hearts of the infected mice in the EV pretreatment group stained with hemoxylin and eosin (H&E) 15 days after infection. The dye showed that, compared with those in the control group mice, the hearts of the EV pretreatment group mice showed a higher parasite load and a wider area of inflammation (Trocolitorrecilhas et al., 2009), suggesting that the EVs may enhanced T-cell infiltration. NO is an important mediator of *T. cruzi* killing activity, and the increased expression levels of IL-10 and IL-4 mRNA can aggravate cardiac parasitic infection in animals. Because they can reduce the level of NO in the heart tissue of *T. cruzi-*infected mice and increase the expression of IL-10 and IL-4 mRNA, EVs may promote parasitism.

In conclusion, the various results of *T. cruzi* studies are largely caused by the complex interaction of hosts and parasites. These interactions include the protein and DNA components in *T*EVs that stimulate different target cells and cause the release of different kinds of cytokines (proinflammatory or anti-inflammatory factors) or NO. To treat Chagas disease most effectively, determining the best way to maintain this delicate immune factor balance is an important goal that will benefit hosts.

### 2.2 Leishmaniasis–*Leishmania*

Leishmaniasis is a zoonotic disease mainly caused by *Leishmania* (Taslimi et al., 2018).The leishmaniasis is widely distributed, but it mainly affects poor areas and developing countries (Ghorbani and Farhoudi, 2017). According to the WHO, an estimated 700,000 to 1 million new cases are diagnosed annually (https://www.who.int/). The disease is mainly transmitted by sand flies (Copa et al., 2019), and its main clinical manifestations can be grouped into visceral leishmaniasis, cutaneous leishmaniasis and mucocutaneous leishmaniasis (De Brito et al., 2018).

#### 2.2.1 Characterization of *Leishmania* extracellular vesicles

In 2010, Silverman et al. (2010b) found that *Leishmania donovani* (*L. donovani*) secreted EVs. Moreover, similar to that in *T. cruzi*, the production of *L. donovani* exosomes is closely related to temperature changes. Studies have shown that the level of exosomes collected from parasites at 37°C is twofold greater than that collected at 26°C (Silverman et al., 2010a). In addition, the expression level of heat shock protein (HSP) was not obviously different between the exosomes produced at pH 7.5 and those produced at pH 5. Moreover, studies have shown that long-term maintenance under *in vitro* conditions does not seem to affect the release of EVs or their immune-related characteristics in mice (Dupin et al., 2021).

#### 2.2.2 Effects of extracellular vesicles on leishmaniasis

*Leishmania* has two cell stages, promastigote and amastigote, in its life cycle. In mammals, promastigotes contact innate immune cells in the mammalian host. To ensure successful infection and reproduction, parasites need to regulate the immune system to inhibit the parasiticidal function of neutrophils and phagocytic monocytes they encounter.

Many researchers have tested the effects of *Leishmania* exosomes on host immune responses (Silverman et al., 2010b; Barbosa et al., 2018; Nogueira et al., 2020). *In vitro*, EVs released by the *Leishmania amazonensis* (*L. amazonensis*) promastigote increased the expression of NO, TNF-α, IL-6 and IL-10 in human monocyte THP-1 cells (Nogueira et al., 2020). Similarly, the EVs released by *L. amazonensis* promastigotes induced the increased expression of IL-6 and IL-10 in macrophages incubated with mouse bone marrow-derived macrophages (BMDMs). However, exosomes from *L. donovani* have also been shown to regulate human monocytes by promoting IL-10 expression and inhibiting TNF-α expression (Silverman et al., 2010b). In addition, mice (pretreated with *L. donovani* exosomes) were infected with *Leishmania* produced more IL-10, IL-4 and CD4+ T cells, leading to the exacerbation of related skin inflammation. In addition, mice pretreated with 15 μg of wild-type parasite exosomes exhibited an 8-fold increase in parasite load compared with that in the control group (Silverman et al., 2010b). The results of these studies show that exosomes are mainly conducive to promoting an immunosuppressive state in a host, allowing the parasite to better reproduce in the infected host. Although there is no evidence that *Leishmania* exosomes contain PGE2 similar to *T. cruzi* EVs, which suppresses the immune response in a *T. cruzi*-infected host, the cause of immunosuppression in *Leishmania* infection may be related to the types of proteins contained in the parasitic EVs.

GP63 (478 amino acid residues) is a conserved protein glycoprotein that is highly expressed on the surface of promastigotes (Razzazan et al., 2008). It is also a virulence factor that facilitates *Leishmania* invasion into macrophages and is considered an important antigen recognized by serum antibodies in patients (Chan et al., 2021). In many studies, patient antigens have been used as DNA vaccine or recombinant protein vaccine in combination with an adjuvant because these treatments can cause a strong Th1 response and offer good immune protection (Sachdeva et al., 2009; Hashemzadeh et al., 2020; Abdellahi et al., 2022). Studies have shown that *Leishmania* virulence factors usually target macrophage protein tyrosine phosphatase (PTP) for cleavage, thus regulating JAK/STAT signaling pathway activation (Hallé et al., 2009; Oliveira L. G. et al., 2022). For example, the cleavage of SHP-1 by GP63 leads to dephosphorylation of JAK-2 and inactivation of the JAK/STAT-1 signaling pathway. Moreover, exosomes collected from GP63^−/−^ *Leishmania major* (*L. major*) failed to regulate PTP expression in a manner similar to GP63^+/+^ *L. major*, confirming the important role played by GP63 in *Leishmania* EVs.

In addition, casein kinase 1 (CK1), a multifunctional Ser/Thr protein kinase family member, plays a very important role in EVs. Studies have shown that inhibition with CK1 reduced the growth of promastigotes (Baker et al., 2021). There are mainly six CK1 subtypes in *Leishmania*, among which CK1.2 is the most studied in *Leishmania* EVs. This kinase is usually found in the extracellular environment, where it can directly phosphorylate extracellular substrates, but it can be exported to infected host cells through the release of exosomes by parasites. Through the phosphorylation of cell host proteins, CK1.2 contributes to *Leishmania* survival in host cells (Smirlis et al., 2022).

In addition, recent studies have shown that the *Leishmania* GAPDH (*Lm*GAPDH) protein is highly enriched in EVs secreted during infection, and GAPDH can inhibit the expression of TNF-α in a host (Das et al., 2021). When *Leishmania* (containing mutant GAPDH) and its EVs were used to infect macrophages, ELISA, RT–PCR and Western blot data indicated that the protein expression of TNF-α increased significantly, but the TNF-α mRNA expression remained unchanged. Moreover, *Lm*GAPDH mediated TNF-α inhibition in a concentration-dependent manner.

Notably, in addition to parasite components, the components of the viruses carried by a parasite, such as *Leishmania* RNA virus 1 (LRV1), likely affect the development of certain diseases. For example, Lamberts confirmed LRV1 particles in 30% of LRV1 + *Leishmania (Viannia) guyanensis* (Lg21) EVs. These EVs promoted the rapid infection of *Leishmania* and showed greater virulence than the *Leishmania* parasites in the *Viannia* complex (Dong et al., 2019). Importantly, Lg21 EVs have been shown to significantly increase the severity of infection, while LRV1 cannot induce the same increased virulence, suggesting that LRV1 requires exosome encapsulation to induce host immunopathology (Atayde et al., 2019). Carvalho showed that EVs containing LRV1 induced TLR-3 activation and NLRP-3 inhibition (de Carvalho et al., 2020).

In conclusion, the role played EVs in leishmaniasis is mainly related to the proteins contained in the EVs (such as GP63, CK1 and GAPDH). Moreover, viruses in *Leishmania* EVs also play important roles in the occurrence and development of diseases; however, the effects of these viruses have not been reported for other kinds of parasites. In general, the role played by EVs is facilitation of parasitization in host cells.

### 2.3 Giardiasis–*Giardia*

Giardiasis, a disease characterized by diarrhea, seriously endangers the health of the humans and other animals (Hajare et al., 2022). Currently, the control of giardiasis depends mainly on the use of anti-*Giardia* drugs, such as metronidazole and tinidazole. However, frequent use of anti-*Giardia* drugs can lead to drug resistance and side effects (Krakovka et al., 2022). However, is no ideal vaccine, mainly because of a lack of understanding of the pathogenesis and regulatory mechanisms in *Giardia* infection.

#### 2.3.1 Characterization of *Giardia* extracellular vesicles

In 2017, Ingrid found that *Giardia* secreted a set of vesicles with different sizes and shapes, including an exosome set with a diameter of 50–100 nm and a group of small vesicles with a diameter of 20–25 nm (Evans-Osses et al., 2017). The release of EVs has been reported to be typically related to cellular responses to various forms of activation: chemical, biochemical and mechanical triggering in a natural or artificial manner. Different pH values significantly affect not only the physiological morphology of *Giardia* but also the release of EVs. Studies have showed that the levels of EVs released at pH 3 within a short time (5–10 min) were significantly higher than those released at pH 8. The change in pH from 5 to 7 in long-term culture (24–48 h) increased the levels of released EVs, but the levels decreased significantly when the pH was increased to 8 (Evans-Osses et al., 2017). In addition, inducers exerted a profound impact on EV production. In a short-term culture, 5% normal human serum (NHS) and 10× bile medium induced a significant increase in the EV production rate compared with cultures without an inducer. Long-term culture or the addition of calcium led to results similar to those obtained in the short-term culture.

#### 2.3.2 Effects of extracellular vesicles on giardiasis

During the life cycle, parasites appear in two forms: 1) trophozoites, which attach to intestinal epithelial cells and reproduce by bisection, and 2) cysts, the infective forms, which are released through feces and internalized through ingestion of infected food or water (Solaymani-Mohammadi, 2022). To ensure successful infection and reproduction, a parasite must adhere to intestinal epithelial cells. However, host immune cells stimulated by EVs can produce corresponding responses that resist the invasion of parasites.

To examine the role of EVs in the production of proinflammatory cytokines induced by *Giardia*, different concentrations of EVs and parasites were incubated with peritoneal mouse macrophages. The following analyses showed that in the EVs group, the highest EVs concentration level was 1.5-fold greater than its lowest concentration level, but it was never as high as the EV concentration in the parasite group. When comparing the effects of the EVs on mouse peritoneal macrophages at different times (18 and 24 h), the results showed that the expression of cytokines such as IL-1β, TNF-α and IL-6 was time-dependent and EV dose-dependent (Pu et al., 2021), indicating that EVs played an important role in the induced inflammatory response of the macrophages.

In terms of signaling pathway activation, studies have shown that EVs can regulate the innate immunity of host cells through the inflammatory TLR2 and NLRP3 signaling pathways in the body. The MAPK, AKT, and NF-κB pathways are involved in regulating the proinflammatory immune response of mouse macrophages *in vitro* (Zhao et al., 2021a; 2021b). Experiments were performed with low and high concentrations of EVs and *Giardia* coincubated with macrophages, and the results showed that the expression levels of NLRP3 and TLR2 were EV dose-dependent (Zhao et al., 2021a). The highest expression was found in the parasite group, which may have been results of worm-specific and combined effects of EVs secreted by the worm on cells in later parasitic stages.

In addition, in the study, the roles played by the MAPK and AKT signaling pathways in EV-induced proinflammatory cytokine production were examined. After inoculating EVs into mouse macrophages, protein phosphorylation in the MAPK and AKT signaling pathways was detected at different times (Zhao et al., 2021b). The results showed that the level of p38 phosphorylation peaked 2 h after EV inoculation and began to decrease 4 h after inoculation. The ERK phosphorylation level increased with the inoculation time and peaked at 4 h. The AKT phosphorylation level reached the highest level at 4 h. These results showed that the p38, ERK and AKT signaling pathways were activated after EVs were inoculated into mouse macrophages. In addition, p38, MAPK and ERK inhibitors showed positive feedback regulation, while AKT inhibitors showed negative feedback regulation.

In the classical signaling pathway, NF-κB p65 is inhibited by a natural inhibitor of NF-κB, IκB. The stimulation of an antigen receptor can activate the IκB kinase (IKK) complex, which leads to IκB phosphorylation. IκB phosphorylation triggers autoubiquitination and degradation, resulting in the release of p65. After protein modification, activated p65 is transported to the nucleus and induces the expression of target genes (Huang et al., 2022). To this end, in the development of giardiasis, EVs may induce the activation of the classical NF-κB signaling pathway through the phosphorylation of IκBα, IKKα and IKKβ, which causes NF-κB p65 translocation to the nucleus. This may further cause changes in the expression NF-κB-regulated cytokines such as IL-1β, IL-6 and TNF-α.

Moreover, parasite adhesion is very important for parasitism. Exosomes released by certain kinds of parasites, such as *Trichomonas vaginalis* (*T. vaginalis*), incubate with highly adhesive parasite strains incubated with parasite strains with low adhesion capacity can increase the adhesion rate of the strains, which can facilitate *T. vaginalis* infection of vaginal and prostate epithelial cells (Twu et al., 2013). To study the role played by *Giardia* EVs in parasite adhesion, Caco-2 (human colon adenocarcinoma cell) cells were used in an adhesion test. EVs treated with protease K at 37°C for 1 h and heat inactivated for 10 min at 95°C were used as the control cells in the adhesion test. The treated EVs showed no effect on the attachment of trophozoites to Caco-2 cells. Then, 1x10^6^ *Giardia* and Caco-2 cells were incubated for 1–3 h in the presence of different concentrations of EVs, and the results showed that when the parasite and host cells were incubated with 7 µg of EVs, the number of *Giardia* trophozoites in the culture medium decreased by 40% within 1 h, in contrast to that in medium without EVs, suggesting that the number of parasites adhering to Caco-2 cells was increased. In addition, EVs released from trophozoites were more conducive to parasites adhesion to Caco-2 cells than those released from cysts (Evans-Osses et al., 2017).

In addition, by treating *Giardia* trophozoites with the PAD inhibitors CL-amidine and cannabidiol (CBD), the production of *Giardia* EVs was significantly reduced in both treatment groups. Specifically, the total protein content of the EVs in the high- and low-concentration CL amidine groups decreased by more than 80% and 70%, respectively. The total protein content of the EVs in the high- and low-concentration CBD groups decreased by more than 70% and 60%, respectively. In addition, the ability of cells to block host pathogen interactions after treatment with EV inhibitors was evaluated. The results showed that both CL-amidine and CBD reduced the rate of trophozoite adhesion to Caco-2 cells. EVs shed from parasites restore the adhesive phenotype after CL-amidine treatment in a dose-dependent manner. The treatment of cells with chelating agents and WTN (a PI3K inhibitor) did not affect trophozoite adhesion (Gavinho et al., 2020).

These results suggest that the effects of EVs on *Giardia* may be complex. On the one hand, parasite-stimulated hosts trigger a series of reactions, including activation of different inflammation-related signaling pathways. On the other hand, to successfully reproduce in the host, parasites release EVs to facilitate better adherence to host cells.

### 2.4 Trichomoniasis—*Trichomonas*

*T. vaginalis* is a common sexually transmitted extracellular parasite. *T. vaginalis* trophozoites reproduce by vertical dichotomy. Trichomonas infection can cause vaginitis and urethritis and increase the risk of cervical cancer, prostate cancer and other diseases, such as HIV (Schumann and Plasner, 2021). Trichomoniasis is the most common nonviral sexually transmitted infection in the world, with approximately 160 million new cases every year (Molgora et al., 2021).

#### 2.4.1 Characterization of *Trichomonas* extracellular vesicles

In 2013, Patricia isolated EVs from *T. vaginalis* through ultracentrifugation of growth medium, which contained 50–100-nm exosomes (Twu et al., 2013). EVs can deliver their contents to host cells. In 2017, Yesica reported the identification of MV-like structures released by *T. vaginalis.* Although these MVs are not often observed in parasites grown in conventional medium, a 9-fold increase in the MVs on the cell surface was observed when they were incubated with CaCl_2_. The protein analysis showed that 18% of the MVs were enzymes related to the metabolism of *T. vaginalis*, 13% were ribosomes, 10% were proteins involved in signal transduction, 7% were cytoskeletal components, and the other 7% were involved in protein transport (Nievas et al., 2018).

Endocytic sorting complex required for transport (ESCRT) has been widely considered to regulate and guide specific molecules into the ILVs in MVBs (Henne et al., 2011; Juan and Fürthauer, 2018). Four major ESCRT complexes are critical for the delivery of ubiquitinated proteins for lysosomal degradation and protein recovery (Coceres et al., 2021). Studies have shown that overexpression of VPS32, a member of the ESCRT complex, can increase the secretion of EVs and the adhesion of parasites to host cells by 33-fold. In addition, since temperature can affect the uptake of EVs by host cells, whether EVs overexpressing vps32 produce corresponding changes to uptake was analyzed. The results showed that when NHPrE cells are incubated at 37°C with EVs isolated from *Tv*VPS32, the uptake of the EVs was increased (Salas et al., 2021).

In addition, VPS32 can affect the types of proteins carried in EVs. On the basis of label-free quantitation (LFQ) intensities, 36 proteins were shown to be sequentially expressed between the VPS32 and control groups, and the expression of 29 of these proteins was upregulated by more than twofold that in the VPS32 EV samples. In contrast, the expression of 7 proteins in the VPS32 EVs was downregulated by more than twofold compared with that in the control groups (Salas et al., 2021).

#### 2.4.2 Effects of extracellular vesicles on trichomoniasis

Studies have shown that EVs play roles in host immunity. However, the roles played by viral endosymbiosis in EVs is not clear. The components of viruses carried by some parasites, such as LRV1, have been shown to affect the development of diseases. Studies have shown that EVs in *T. vaginalis* infected with a virus are engaged in symbiotic activity with the virus, serving as carriers of intercellular communication mediators and modifying the EVs-carried proteins to inhibit host immune activation, which benefits protozoan parasites (Govender et al., 2020).

Studies have shown that incubation with EVs secreted by *T. vaginalis* (*Tv*EVs) can stimulate RAW264.7 cells to express IL-10 (an anti-inflammatory cytokine) (Olmos-Ortiz et al., 2017). Moreover, expression in macrophages was found to increased more than 15-fold. In addition, to explore the effects of *Tv*EVs on infected female mice, a TvEV-pretreated group (50 μg/ml *Tv*EVs were administered 2 days before parasite inoculation), parasite-infected group and control group were established during the study. The analyses showed that the content of IL-10 in vaginal washes increased significantly on the 8th and 16th days after infection, and the production of IL-17, IL-6 and IL-13 and vulvar inflammation decreased significantly. The results showed that *Tv*EVs exerted an immunomodulatory effect on parasite-induced cytokine expression and promoted a reduction in the inflammatory process in mice infected with *T. vaginalis*.

*Tv*GP63 (homologous to a *Leishmania* virulence protein) and TvTSP8 (tetraspanin) have been found in *Tv*EVs (Twu et al., 2013; Nievas et al., 2018). *Tv*TSP8 is a transmembrane protein located on the surface and in intracellular vesicles and is related to cell adhesion, migration and proliferation in vertebrates. The expression levels of endogenous *Tv*TSP8 in four parasite strains (highly adhesive strains and strains with low adhesiveness) were analyzed. The *Tv*TSP8 mRNA levels in the strains with high adhesiveness were eight- and 10-fold higher than those of the strains with low adhesiveness, which showed that the mRNA expression of *Tv*TSP8 is closely related to adhesion. As a necessary protein for *T. vaginalis*, *Tv*GP63 primarily induces host cell cytotoxicity, not increased parasite adhesion. Studies have shown that *Tv*GP63 protease plays an important role in *T. vaginalis* infection. 1,10-Phenanthroline, a specific inhibitor of *Tv*GP63 protease, significantly inhibited the destruction of HeLa cells by parasites, while EDTA, another chelating agent, did not exhibit this inhibitory effect (Ong et al., 2022).

In conclusion, the effects of EVs on the host are complex. EV secreted by *T. vaginalis* cause the expression of a large number of anti-inflammatory factors in host cells. Although EVs reduce the inflammatory response caused by parasitic infection, they also diminish the killing effect of the host on the parasite in a disguised way. In addition, parasite EVs have the ability to promote parasite adhesion. Therefore, EVs may be conducive to parasitism.

## 3 Phylum sarcomastigophora, class lobosea

### 3.1 Amebiasis—*Entamoeba*

The protozoan parasite *Entamoeba histolytica* (*E. histolytica*) causes amebiasis, which is a major health problem in developing countries (Li J. et al., 2021). *E. histolytica* presents in two stages: trophozoite and cyst. When cysts are ingested with contaminated food or water, they reach the small intestine. After excystment, trophozoites divide longitudinally in the small intestine, sometimes causing tissue destruction and invasive diseases invasion into the colon or liver. When the surrounding environment changes, trophozoites can be transformed into cysts, excreted into the environment through feces and infect new hosts (Carrero et al., 2020).

#### 3.1.1 Characterization of *Entamoeba* extracellular vesicles

In 2020, Sharma et al. (2020) found that *E. histolytica* can secrete EVs. After analyzing serum-free trophozoite culture medium, this group showed that EVs secreted from *E. histolytica* constitute a group with an average diameter of 125 nm (Sharma et al., 2020). In addition, experiments revealed that the protein composition of the EVs in different life stages differed. EVs isolated from the cyst parasite promoted encystment, while EVs from the trophozoite hindered encystment.

To detect the protein composition of these EVs, five independent replicates of an experiment was carried out. A total of 719 different proteins were thus identified, and coat protein Ⅰ vesicle shell, the proteasome core complex and the Arp2/3 protein complex were highly enriched in the samples. In contrast, these EVs did not contain complete components of a cell membrane or the histone acetyltransferase complex (Sharma et al., 2020). In contrast to those shed by *T. vaginalis*, EVs secreted by *E. histolytica* did not contain tetratransmembrane proteins. Moreover, amoeba EVs selectively carried certain small RNAs.

#### 3.1.2 Effects of extracellular vesicles on amebiasis

The pathogenesis of parasitic infection is closely related to the regulatory mechanism of parasite gene expression. Studies have shown that *E. histolytica* triggers an interfering RNA (RNAi) mechanism, and the Argonaute 2–2 (*Eh*Ago 2–2) protein is carried by EVs. An analysis of *Eh*Ago 2-2 showed that *Eh*Ago 2-2 interacts with at least 43 proteins, and the pathways of its interacting proteins involve small RNA (sRNA) loading, RNA binding, vesicle transport for subsequent protein folding and modification, signal transduction and so on (Foda and Singh, 2015). Another key protein carried by EVs is the tudor staphylococcal nuclease (TSN) protein. Analyses have shown that at least 32 proteins interact with TSN. These TSN interactors are involved in transcription, stress response, signal transduction and metabolism, as well as other cellular processes. Notably, TSN can bind to upstream regulatory element 1 (URE1), which is related to an amoeba virulence protein. It has been suggested that TSN plays a role in virulence regulation as a transcription factor (Calixto-Gálvez et al., 2011; Cázares-Apátiga et al., 2017). In addition, after heat shock treatment (42°C for 2 h), TSN colocalized with HSP70 (another protein in *Entamoeba*-shed EVs). In addition, studies have revealed a full spectrum of 27-nt sRNAs related to *Eh*Ago 2–2. A comparative analysis of sRNA populations in toxic and nontoxic amoeba strains showed that sRNA populations may regulate virulence gene expression.

### 3.2 Granulomatous amoebic encephalitis—*Acanthamoeba*

*Acanthamoeba castellanii* (*A. castellanii*) is commonly found in polluted soil and water, and seven pathogenic species have been isolated to date, among which *A. castellanii* is among the most common (Wehelie et al., 2021). The environmental importance of *A. castellanii* as a host and carrier of microorganism (including bacteria, viruses or fungi) is recognized. The invasion route of *A. castellanii* is not completely clear. The parasite is known to enter the human body through skin wounds, damaged ocular conjunctiva and respiratory and reproductive tracts. Most of the *A. castellanii* strains infect the brain, eyes, and skin. Moreover, *A. castellanii* can cause a rare but fatal form of encephalitis, GAE, which is associated with immunologically compromised patients. Due to the lack of specific anti-*Acanthamoeba* drugs and the selectivity of the blood–brain barrier, the mortality of GAE is higher than 90% (Keane et al., 2020; Wang et al., 2021).

#### 3.2.1 Characterization of *Acanthamoeba* extracellular vesicles

In 2019, Lin and others identified EVs secreted by *A. castellanii* (Gonçalves et al., 2019; Lin et al., 2019)*.* They found that small EVs (< 50 nm), medium EVs (50–200 nm) and large EVs (> 200 nm) in the *Acanthamoeba* culture medium. In addition, EVs produced under different culture conditions differed. By comparing the cultures in different media (i.e., 712 PYG and glucose media), Researchers found no significant difference in the average diameter of EVs in these media, but the range of EV variation in the PYG medium was larger than that in the glucose medium. This finding suggested that the difference in EVs in the PYG medium (PYG EVs) was more obvious than that in the EVs in the glucose medium. In addition, nutrient stress exerted a significant impact on the composition of the EVs. Supernatants from PYG and glucose medium cultures were evaluated by mass spectrometry. In these samples, 134 species-specific proteins were found only in the EVs in the glucose medium, and 89 species-specific proteins were found only in the EVs in the PYG medium. A quantitative proteomic analysis of these EVs showed that the largest protein families in the EVs were composed of hydrolases and oxidoreductases (Lin et al., 2019).

#### 3.2.2 Effects of extracellular vesicles on GAE

EVs of parasites typically cause changes in the expression level of inflammatory factors in host cells. Studies have shown that EVs produced by *A. castellanii* can affect the mRNA expression levels of the cytokines IL-6 and IL-12 secreted by human monocyte THP-1 cells. Among these cytokines, the transcription of the proinflammatory cytokines IL-6 and IL-12 was increased from twofold to threefold. After cell exposure to EVs from *A. castellanii*, CXCL10 transcription did not change significantly (Gonçalves et al., 2019), similar to the observations reported for *T. vaginalis* and *Toxoplasma gondii*, proving that EVs from *A. castellanii* deliver information to immune cells and trigger an immune response.

EVs shed from parasites exert a significant impact on a host. Because certain RNAs and proteins in EVs that enter host cells induce various immune reactions, EVs internalization by host cells is very important. Fluorescent labeling revealed that EVs produced by *A. castellanii* were internalized by host epithelial cells (CHO cells), T98G cells in the blood–brain barrier and C6 neuroglia rat cells. In addition, the effect of C6 cell incubated with *A. castellanii* for different durations was evaluated with a cytopathic effect (CPE) assay. The results showed that EVs were observed in C6 cells within 15 min. When C6 cells were incubated with EVs shed by *A. castellanii* for 24 h, the C6 cells were significantly dispersed within the culture plate (Gonçalves et al., 2019), indicating that parasite-derived EVs destroyed the adhesive ability of targeted cells and led to their death.

The CPE on C6 cells treated with *A. castellanii*-secreted EVs may be related to leucine aminopeptidase. An analysis revealed that pretreatment of *Acanthamoeba* secreted proteins (ASPs) with a leucine aminopeptidase inhibitor and a specific antibody against the *A. castellanii* M20/M25/M40 super family aminopeptidase attenuated the cell damage caused by coculturing ASP with cells (Huang et al., 2017). These results suggest that this extracellular aminopeptidase plays an important role in the pathogenesis of *A. castellanii*. According to a proteomic analysis of EVs secreted by *A. castellanii*, several proteins exhibit aminopeptidase activity and may contribute to the pathogenesis induced by EVs.

In conclusion, EVs produced by *A. castellanii* can induce the expression of the cytokines IL-6 and IL-12 in host cells and can damage cells through leucine aminopeptidase carried in the EVs. However, relatively few studies have been performed on EVs produced by *A. castellanii*, and the protein analysis of *A. castellanii* EVs has indicated that these EVs carry certain proteins that have been reported to exhibit important functions in other parasites (such as HSP83 and HSP60); therefore, the impact of EVs-delivered proteins on the occurrence and development of diseases needs to be further explored.

## 4 Phylum apicomplexa, class sporozoasida

### 4.1 Malaria—*Plasmodium*

**FIGURE 1:**
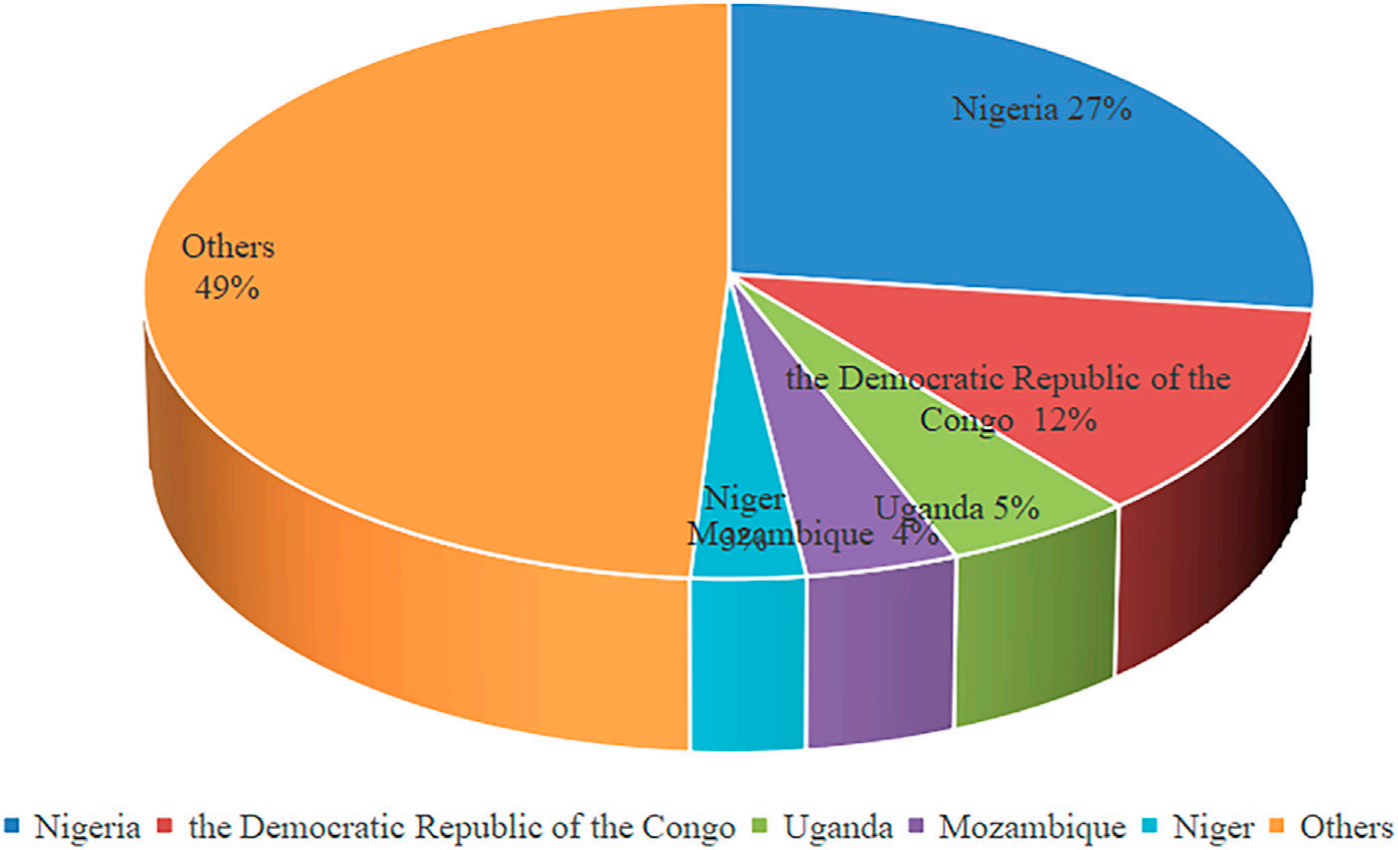
The ratio of people infected with malaria in different countries in 2019.

Malaria is an insect-borne disease caused by *Plasmodium* infection that is caused by the bite of *Anopheles* mosquitoes or the transfusion of blood from *Plasmodium* carriers (Bihoun et al., 2022). More than 3.4 billion people in the world are at risk of contracting malaria, and more than 200 million people were infected with malaria in 2019 **(** https://www.who.int/ **)**. In 2019, five countries accounted for about half of global malaria deaths (Figure 1) Severe malaria syndrome includes mainly severe anemia, metabolic acidosis, multiple organ failure and cerebral malaria (CM) (Idro et al., 2010). The main pathogen causing CM is *Plasmodium falciparum* (Luzolo and Ngoyi, 2019), which is the cause of essentially all malaria-related deaths. The effectors secreted by parasites may play important roles in malaria.

#### 4.1.1 Characterization of *Plasmodium* extracellular vesicles

In 2017, Abdirahman measured EVs obtained by ultracentrifugation of *Plasmodium*-infected samples, and the results showed that the average diameter ± SD of the EVs was 143 nm ± 66 nm, which overlapped the size range of exosomes (30–150 nm) and microbubbles (100–1,000 nm) (Jensen et al., 2020). An analysis of the EV protein components showed that 61 and 149 kinds o*f Plasmodium falciparum* (*P. falciparum*) proteins were identified in *P. falciparum* EVs purified from a ring to trophozoite (RT) medium and from a trophozoite to the next cycle of ring (TR) medium, respectively.

#### 4.1.2 Effects of extracellular vesicles on malaria

Studies have shown that *Plasmodium* EVs can exert an important impact on the occurrence and development of malaria. Since early exosomes and particles were not combined or considered to be EVs, research into exosomes and particles has progressed separately. In 2002, Piguet et al. (2002) found that, although microparticles are found in normal blood, the number of microparticles in plasma containing *Plasmodium berghei* was increased significantly, and the main sources of microparticles were platelets (Piguet et al., 2002). In 2005, Combes et al. (2005) confirmed that *P. berghei* microparticles were the sources of the EVs in plasma and that they showed good procoagulant activity. In 2011, Nantakomol et al. (2011) found that malaria patients showed increased levels of MVs derived from erythrocytes. After antimalaria treatment, these MV levels decreased rapidly (Nantakomol et al., 2011). In 2013, Mantel et al. (2013) found that MVs derived from erythrocytes infected with *P. falciparum* directly mediated communication between parasites through EVs.

Studies have shown that EVs may play roles in immune regulation, nutrient acquisition and *in vivo* pathogen invasion. In 2005, the roles played by *P. berghei* microparticles were reported. ATP binding cassette transporter A1 (ABCA1) significantly alters lipid distribution and accumulation on the membrane and exposes phosphatidylserine (PS) on the outer plasma membrane surface (Peters et al., 2022). Giovanna found that the number of EVs and the procoagulant activity of the plasma in ABCA1^−/−^ mice were significantly reduced compared with those in ABCA1^+/+^ mice. In addition, microparticles derived from the ABCA1^+/+^ mice infected with *P. berghei* (the ANKA stain) induced a significant increase in the amount of TNF released from uninfected macrophages (Combes et al., 2005). This finding suggests that TNF may play a key role in determining the severity of acute malaria by regulating the production of cell-specific MVs.

In addition to the roles played by EV proteins, EV mRNAs play important roles. In the asexual erythrocytic stage, EVs are involved in significant interactions between host erythrocytes and parasites. These interactions lead to the transfer of human microRNA (miRNA) from erythrocytes to parasites. A report showed that, compared with those in normal erythrocytes, high levels of specific human mir-451, mir-223 and let-7i were found in erythrocytes with HbAS (sickle cell trait) and HbSS (sickle cell disease) genotypes and exerted a negative regulatory effect on *Plasmodium* infection, explaining the reason that patients with sickle cell disease are affected to a lesser degree by malaria than people with normal erythrocytes (Muriuki et al., 2021). In addition, studies have shown that RNAs in EVs produced by infected red blood cells (iRBCs) can be successfully internalized into monocytes and macrophages. Moreover, RNAs in EVs ingested by monocytes and macrophages may play different roles (Alfandari et al., 2022).

Many studies have shown that increased release of EVs containing miRNAs by certain cell types, such as mast cells (MCs), is associated with malaria severity (Huang et al., 2021). Intravenous injection of MC exosomes significantly increased the incidence of CM in mice infected with *P. berghei* (ANKA), and they exacerbated liver and brain histopathological damage, promoted the expression level of Th1, and aggravated the activation of the cerebral vascular endothelium and the destruction of the blood–brain barrier in the CM mice. In addition, treatment with MC exosomes resulted in decreased cell viability and mRNA expression levels of Ang-1, ZO-1 and claudin-5 but increased mRNA expression levels of Ang-2, CCL2, CXCL1 and CXCL9, suggesting that MCS exosomes may worsen the pathogenesis of mouse CM.

In summary, the relationship between EVs and disease is reflected in many cells, such as endothelial cells, RBCs, and macrophages. Increasing evidence shows that EVs are closely related to malaria. For example, platelet MVs can bind to *P. falciparum-*iRBCs in a *Pf*EMP1-dependent manner, transfer platelet antigens to the iRBCs, and induce iRBCs to adhere to endothelial cells, thus participating in the development of CM (Melcher et al., 2010). The pathological stimulation of iRBCs in the brain shows that iRBCs can promote their own adhesion. The content carried in EVs is the key to the development of disease.

### 4.2 Neosporosis—*Neospora*

Neosporosis is a disease caused by *Neospora caninum* (*N. caninum*)*,* which parasitizes a variety of animals and is widely distributed throughout the world (Calarco and Ellis, 2020). The main harm caused by this disease is abortion or stillbirth in pregnant animals and motor nerve disorder in newborns. The effect of this disease on cattle is of particular concern because it is the main cause of bovine abortion (Yang et al., 2021). *N. caninum* infection occurs mainly through host intake of food and/or water contaminated by oocysts in canine feces (Li et al., 2014). In addition, placental transmission is considered to be an important route of infection (Gharekhani and Yakhchali, 2020).

#### 4.2.1 Characterization of *Neospora* extracellular vesicles

In 2018, Li et al. found EVs secreted by *N. caninum* by collecting the supernatant of parasite culture medium. In this study, 705 proteins were identified in the EVs secreted by *N. caninum*, and many duplicate proteins were found in the exosomes derived from *N. caninum* and from other protozoan parasites, such as *Trichomonas*, *Echinococcus* and *Leishmania*. Moreover, proteins involved in EVs biogenesis and transport, such as 14-3-3-3, HSP70 and HSP90, as well as membrane-associated antigens, were highly enriched in the purified EVs.

#### 4.2.2 Effects of extracellular vesicles on neosporosis

Innate immune cells, such as macrophages, play crucial roles in controlling the initial parasite replication and pathogenesis stages in neosporosis because these cells contribute to the first line of defense against intracellular infection. After infection with *N. caninum*, various pattern recognition receptors (PRRS) in innate immune cells are activated to induce a series of immune responses to control the proliferation of and infection with *N. caninum*. Because *N. caninum* EVs can transfer antigens to BMDMs, they play important roles in the pathogenesis of neosporosis. Studies have shown that a variety of parasites can be directly transmitted to target cells and thus regulate the immune response through molecules in EVs. After incubating EVs with cells for 2 h, EVs (labeled with green fluorescence) were found in the cytoplasm of BMDMs, and the fluorescence intensity increased steadily (Li et al., 2018). Because *N. caninum* EVs transfer antigens to BMDMs, they play an important role in the pathogenesis of neosporosis.

In addition, similar to the effects of other parasite secreted EVs on the production of inflammatory factors in host cells, the expression levels of IL-12p40, TNF-α, IL-1β, IL-6, IFN-γ and IL-10 in the BMDMs were significantly increased, with the expression of IL-6 and IFN-γ is time-dependent (Li et al., 2018). In addition, when immunized with EVs, the survival rate of infected mice reached 60%, while mice in the control group died within 13 days (Li S. et al., 2021). This suggests a protective effect of the EVs.

## 5 Conclusion

**FIGURE 2:**
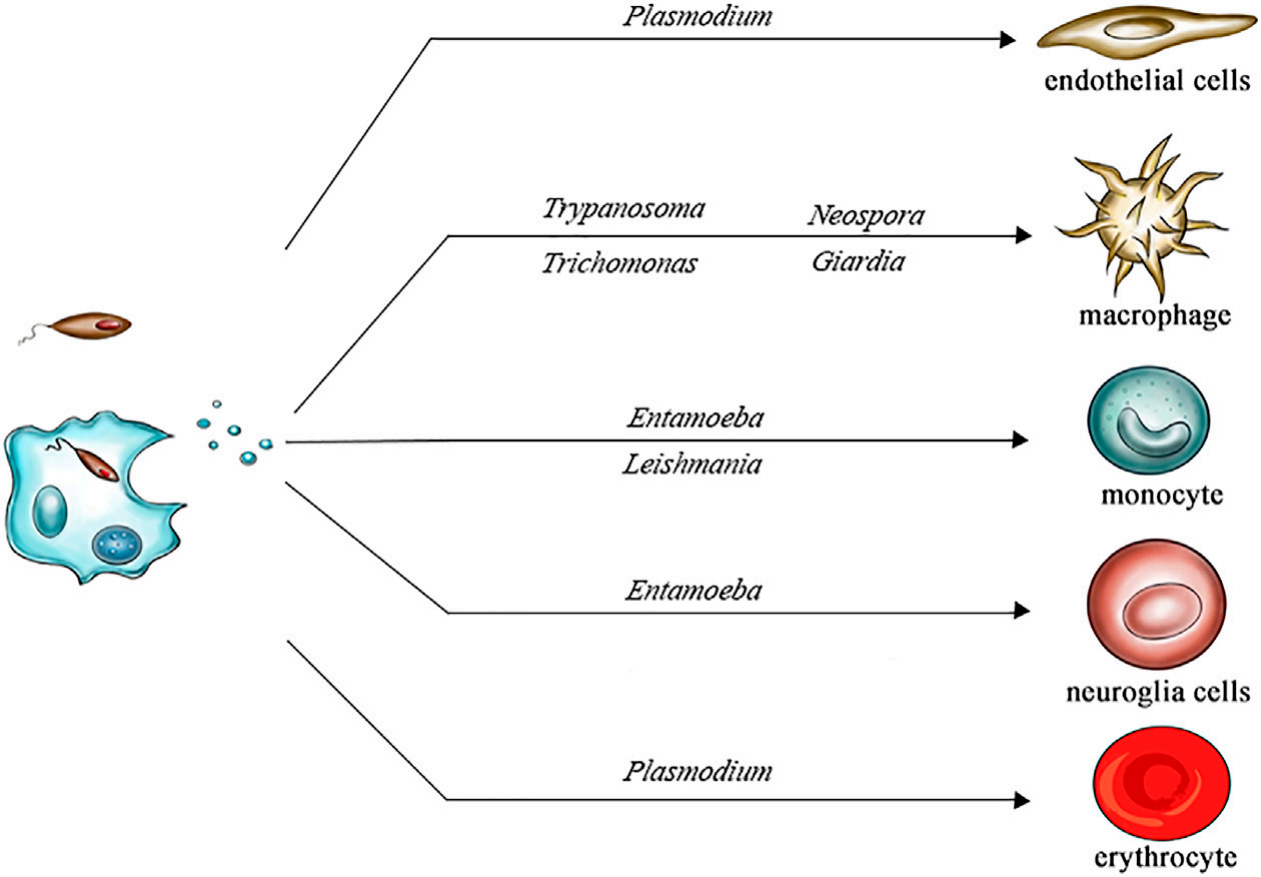
Extracellular vesicles shed by different parasites infect different cells.

In summary, we discussed the effects of EVs secreted by pathogens in several different parasitic diseases, such as chagas disease, leishmaniasis, giardiasis, trichomoniasis, amoebiasis, malaria and neosporosis (Figure 2). For example, GP63 in the EVs released by *Leishmania* lead to dephosphorylation of JAK-2 and inactivate the JAK-2/STAT-1 signaling pathway by stimulating SHP-1 in macrophages. The release of EVs by *Giardia* induced TLR2 and NLRP3 expression in host cells, and the MAPK, AKT and NF- κB pathway and p38, ERK and NF- κB signal pathways were changed. Typically, EVs plays active roles in the immune escape of parasites and parasitic infection, but sometimes the effects of EVs on the host may be complex; for example, the inflammatory factor expression induced by *T. cruzi* in human macrophages and mouse macrophages differs. Therefore, clarifying the effects of EVs on parasites may help to better develop antiparasitic drugs.
